# Supplementary material for: xOmicsShiny: an R Shiny application for cross-omics data analysis and pathway mapping
Source: Bioinform Adv. 2025 May 24;5(1):vbaf097. doi: 10.1093/bioadv/vbaf097 (PMC12117368; doi:10.1093/bioadv/vbaf097)
Supplement: vbaf097_Supplementary_Data [file vbaf097_supplementary_data.pdf]

xOmicsShiny: an R Shiny application for cross-omics data analysis and pathway mapping

Supplementary data

Benbo Gao[1,#], Yu H. Sun[1,#,*], Xinmin Zhang[2], Tinchi Lin[1], Wei Li[2], Romi Admanit[1], and Baohong Zhang[1]

[1]Research and Development, Biogen Inc., Cambridge, MA, USA.

[2]Data Science, BioInfoRx Inc., Madison, WI, USA.

[#]Contributed equally to the work. *To whom correspondence should be addressed.

Table of contents

Figure S1: Modular design of the main functions.

Figure S2: Cross-comparison analysis.

Figure S3: Venn diagram for cross-omics gene and protein comparison.

Figure S4: Comparison result merging.

Figure S5: Gene expression plots with p-values highlighted.

Figure S6: Prize-collecting Steiner Forest (PCSF) network analysis.

Figure S7: Plots generated by the modules related to drug studies.

Figure S8: Geneset Enrichment module.

Figure S9: Pathway visualization.

Figure S10: Overlay of data on the WikiPathways diagrams.

Figure S11: Overlay of data on Metabase pathway diagrams.

Table S1: Running speed test of example datasets.

Table S2: Feature comparison with existing R Shiny applications.

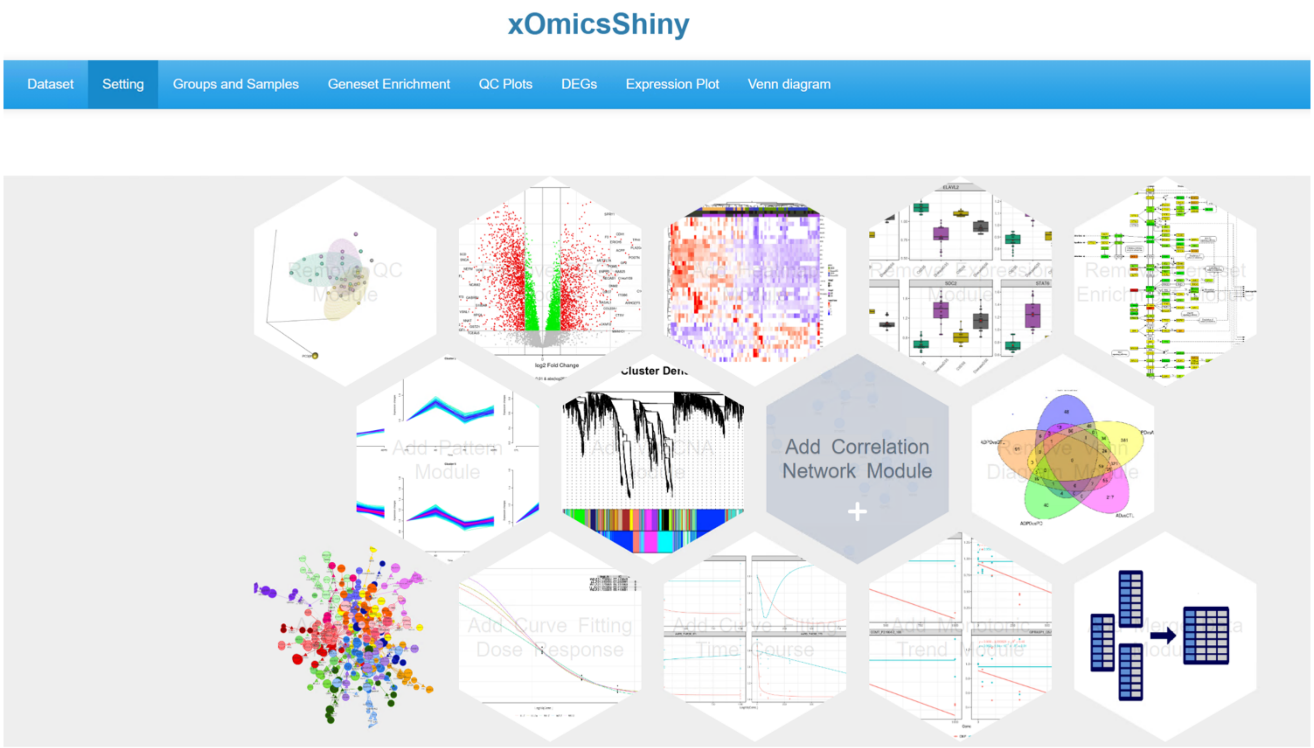

Figure S1: Modular design of the main functions. Users can mouse over a module to add or remove it. The ‘Correlation Network Module’ is selected in the screenshot.

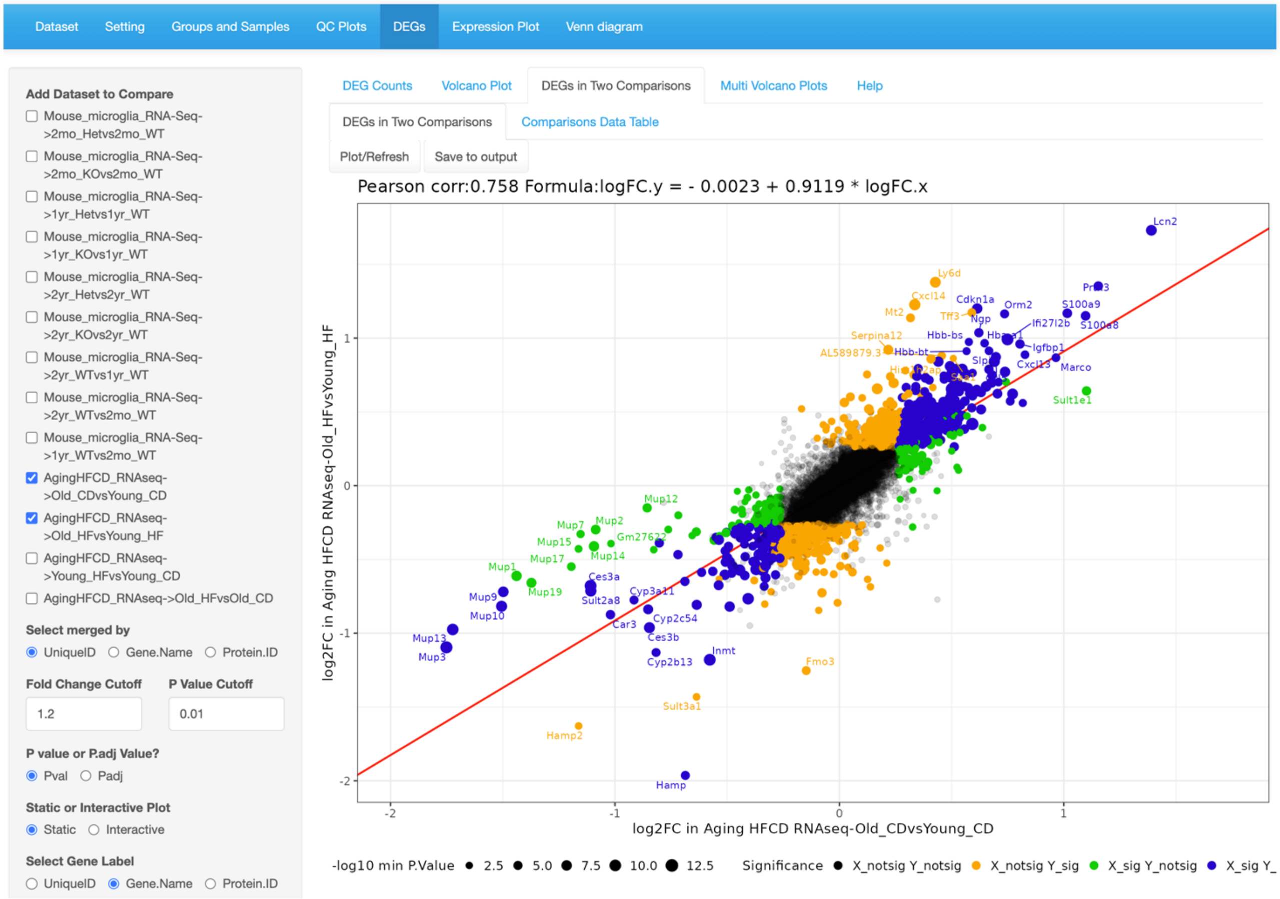

Figure S2: Cross-comparison analysis. Related comparisons can be compared directly using a scatter plot to identify the common and distinct differentially expressed genes (DEGs).

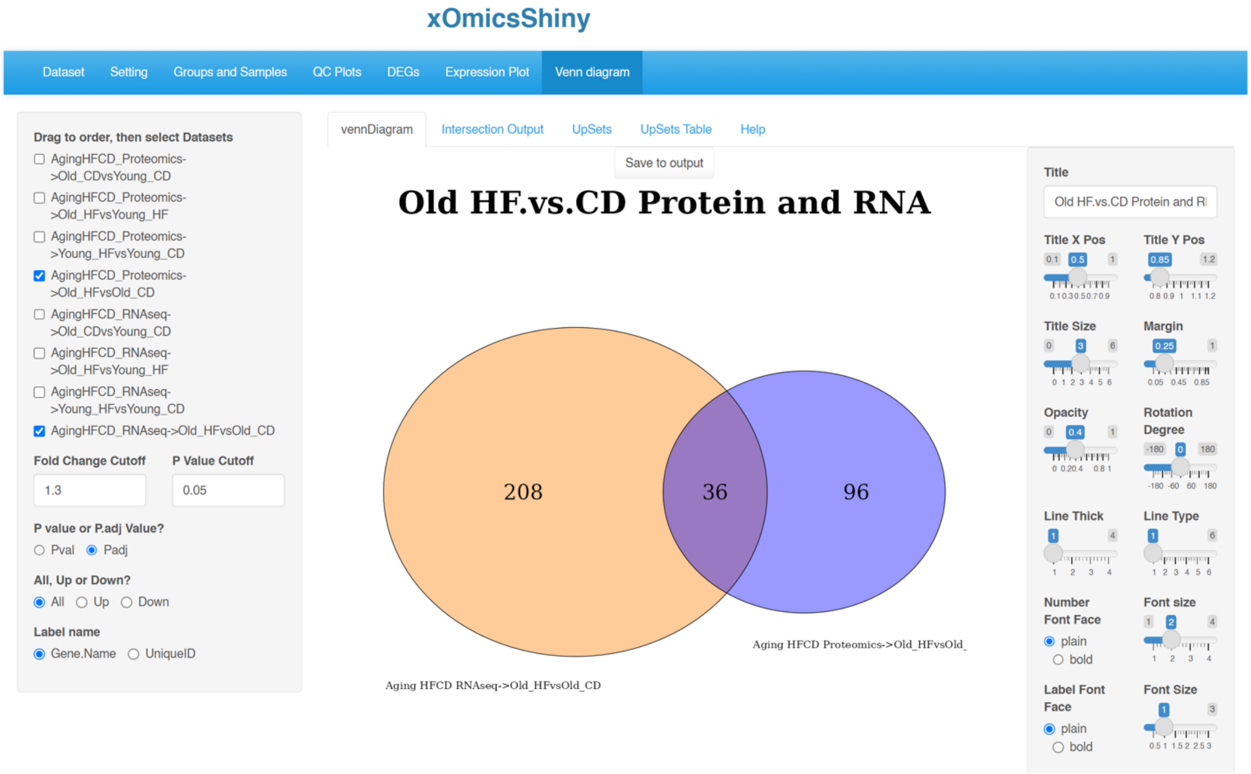

Figure S3: Venn diagram for cross-omics gene and protein comparison. The screenshot shows the overlapping significantly changed genes (left circle) and proteins (right circle) in the Old_HF (old mice, high fat diet treated) group compared with the Old_CD (old mice, control diet treated) group, in RNA-Seq and proteomics.

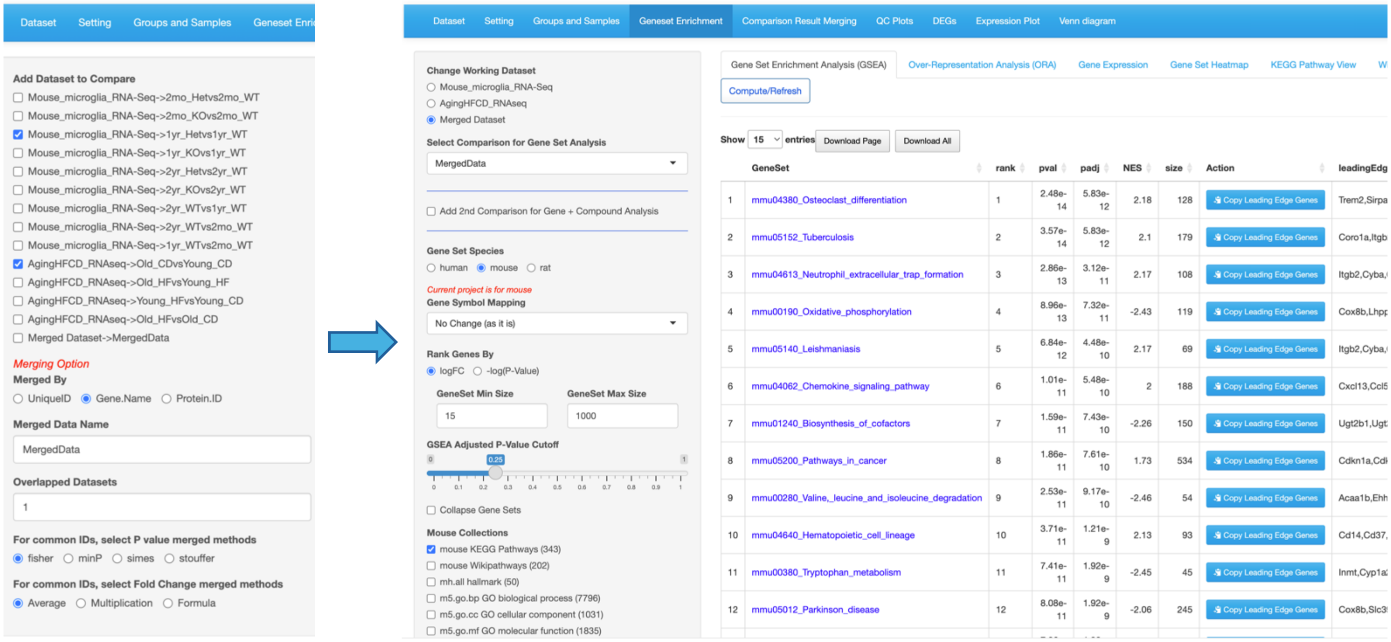

Figure S4: Comparison result merging. Related comparisons from different studies (left panel) can be merged to retain common DEGs, which can be further explored using other modules, such as the Geneset Enrichment module (right panel).

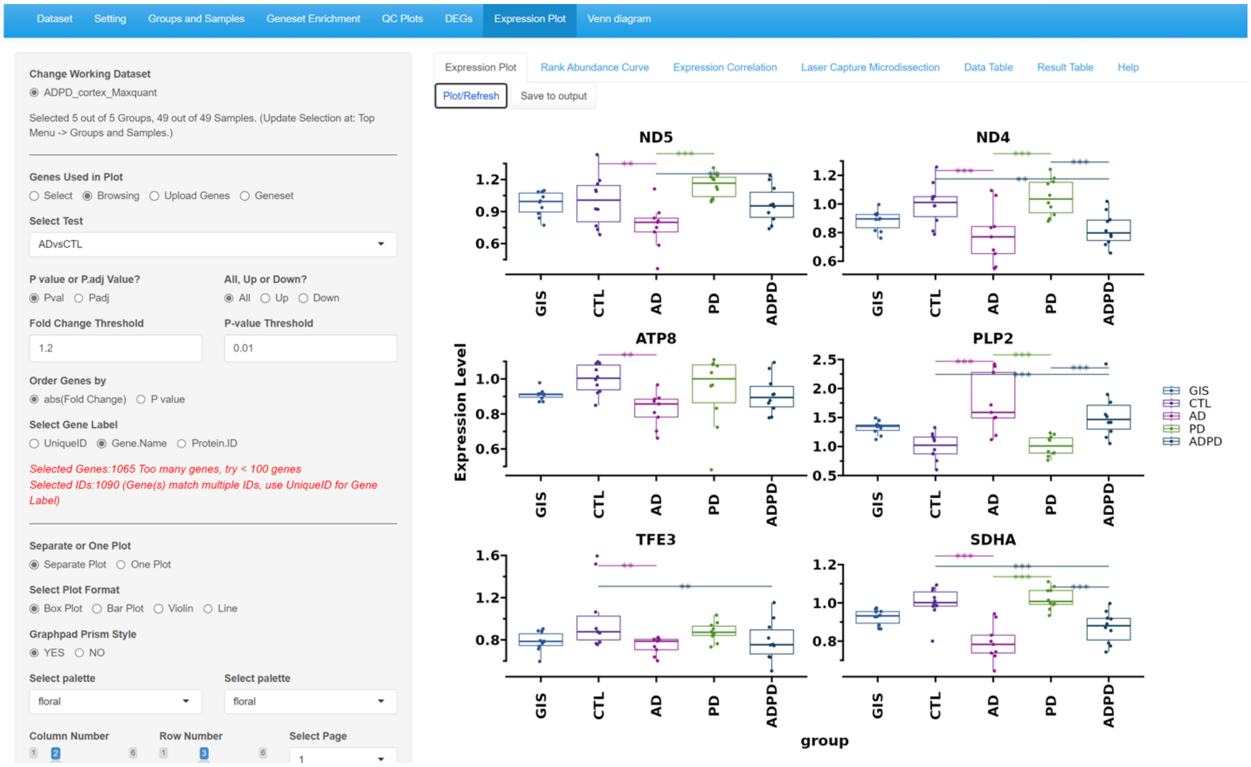

Figure S5: Gene expression plots with p-values highlighted.

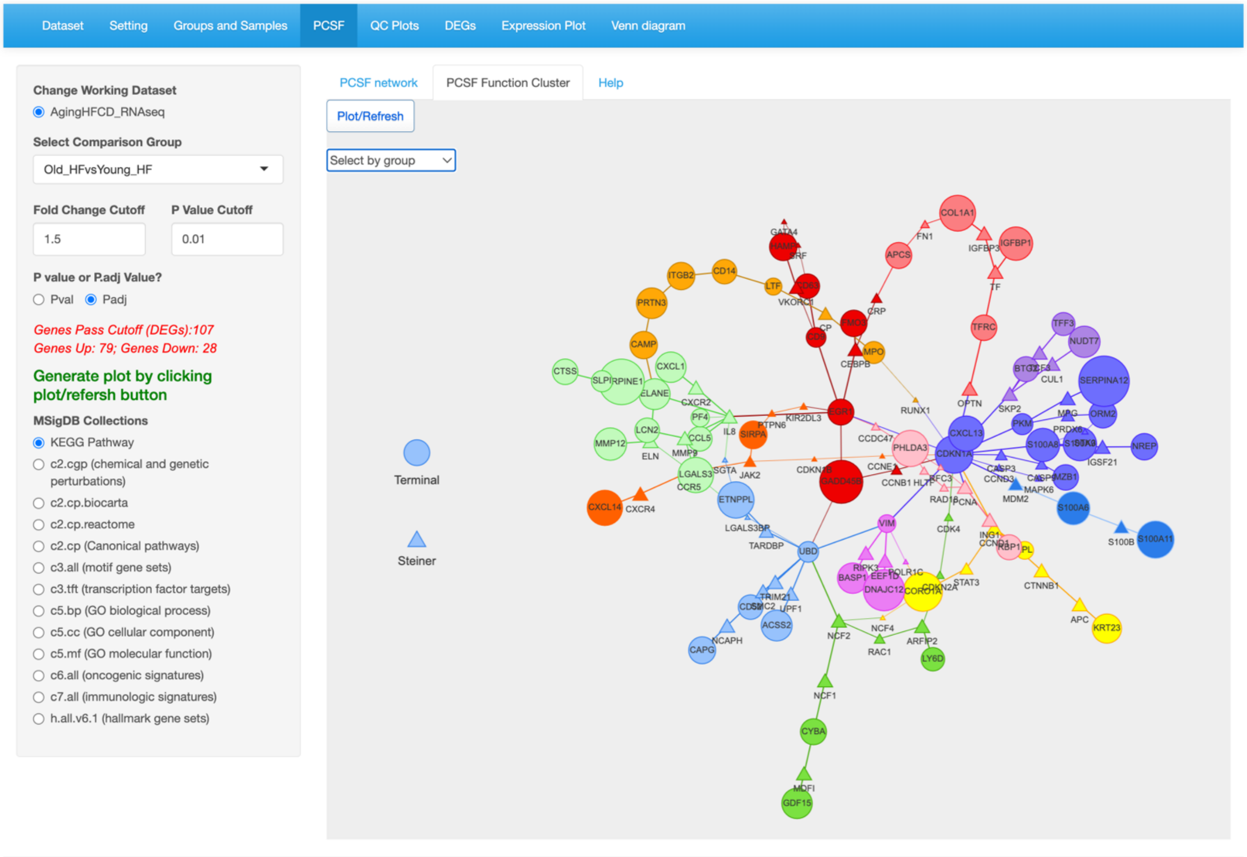

Figure S6: Prize-collecting Steiner Forest (PCSF) network analysis. The PCSF method performs mapping of the data to biological networks, such as protein-protein interactions, to gain further insights into the functional units.

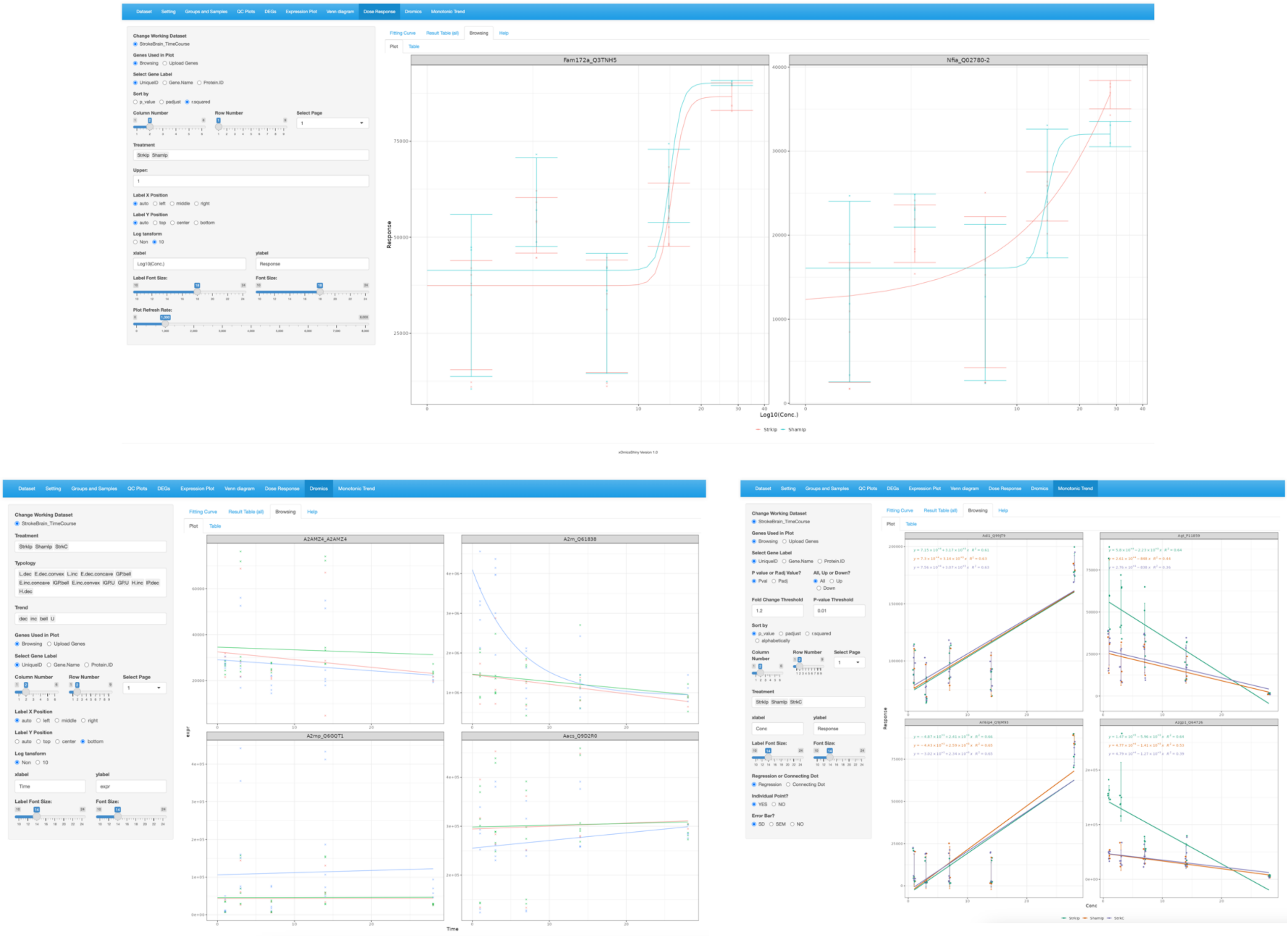

Figure S7: Plots generated by the modules related to drug studies. Dose Response (upper), Time Course (Dromics, lower left) and Monotonic Trend (lower right).

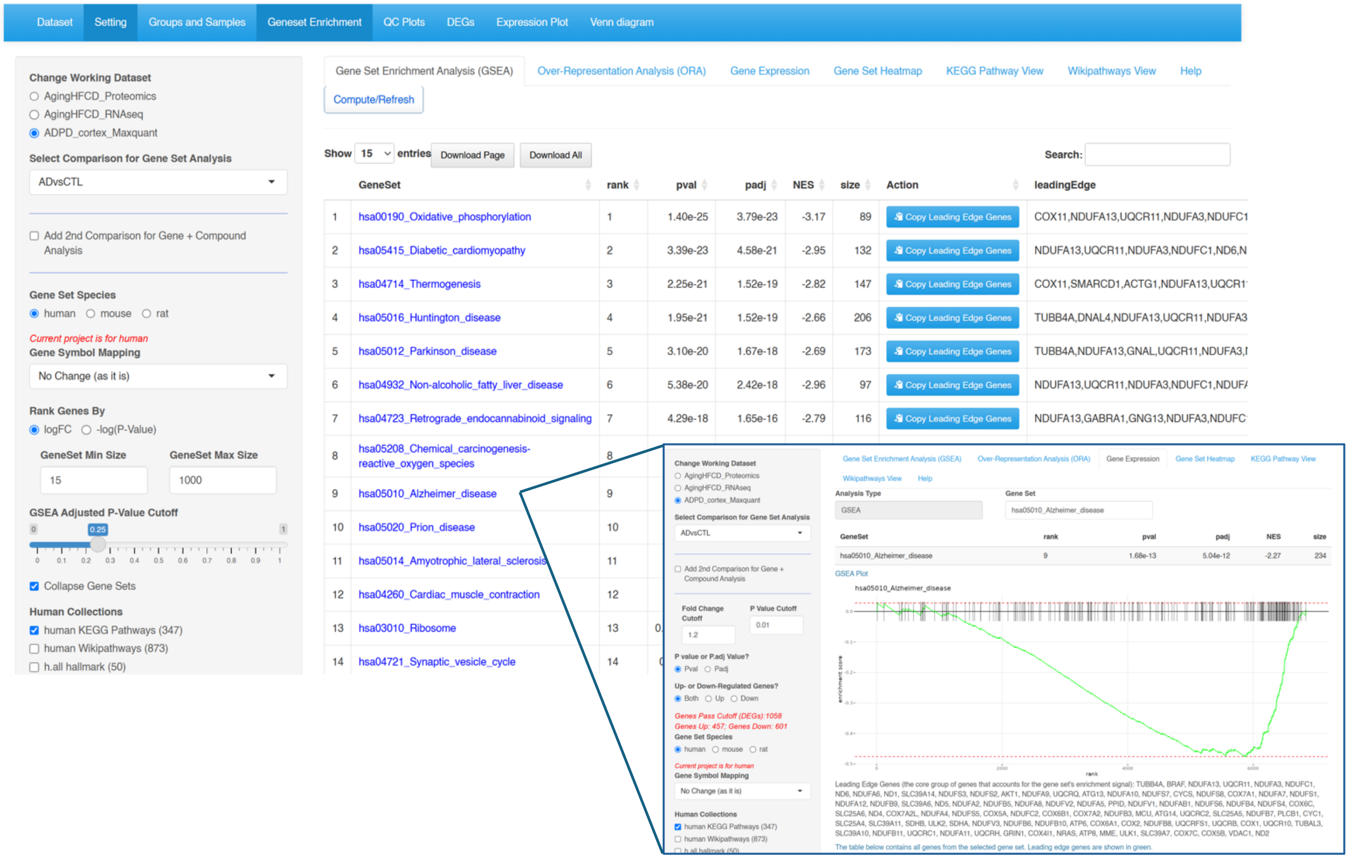

Figure S8: Geneset Enrichment module. Users can further explore the GSEA plots by clicking on the enriched pathway names (using the Alzheimer's Disease pathway as an example).

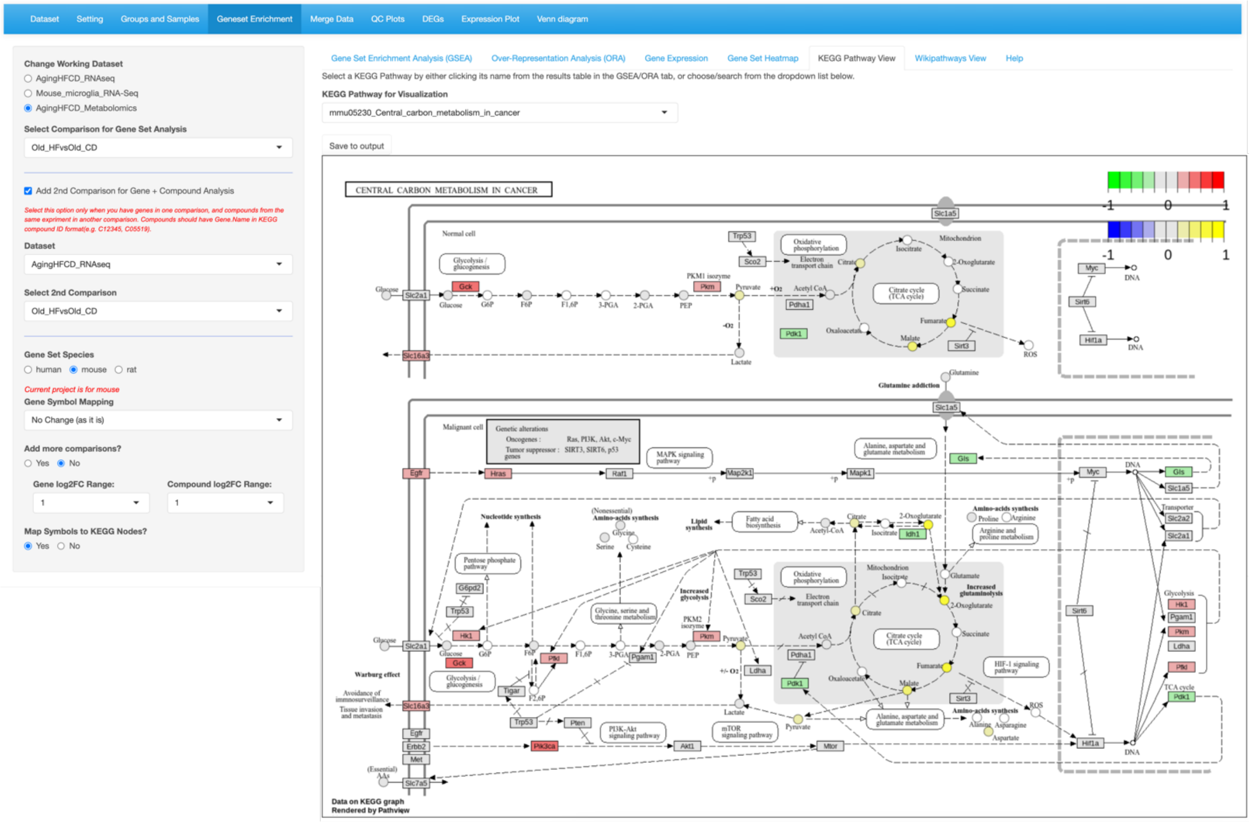

Figure S9: Pathway visualization by mapping genes and metabolites to KEGG pathway maps. Related comparisons from different studies can be merged and compared directly.

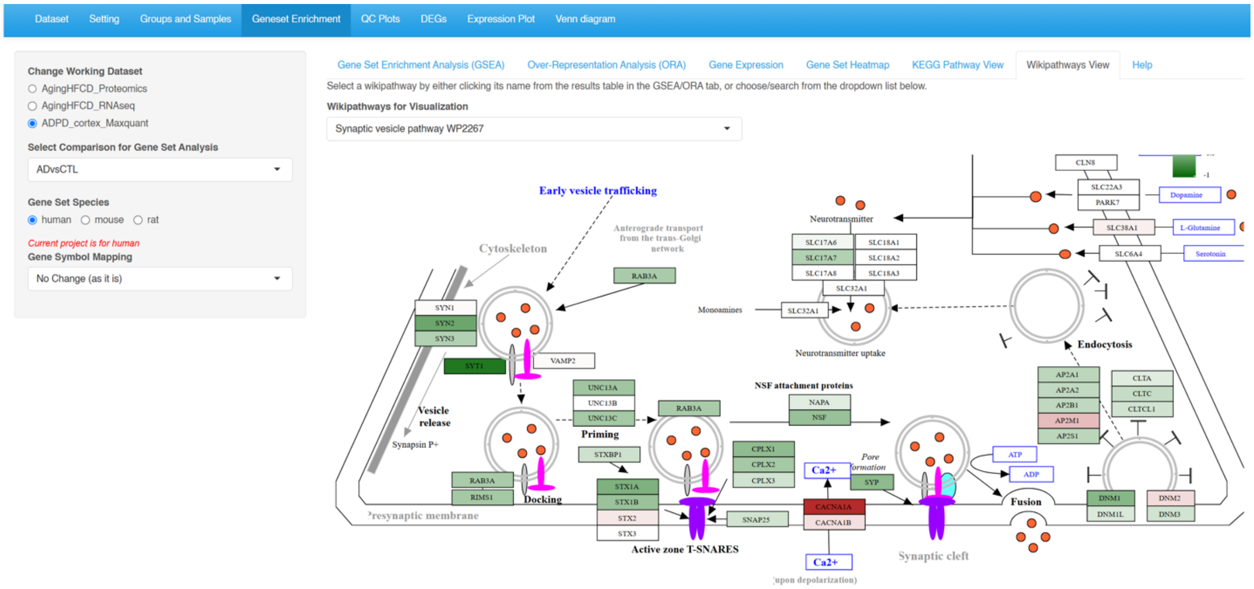

Figure S10: Overlay of data on the WikiPathways diagrams.

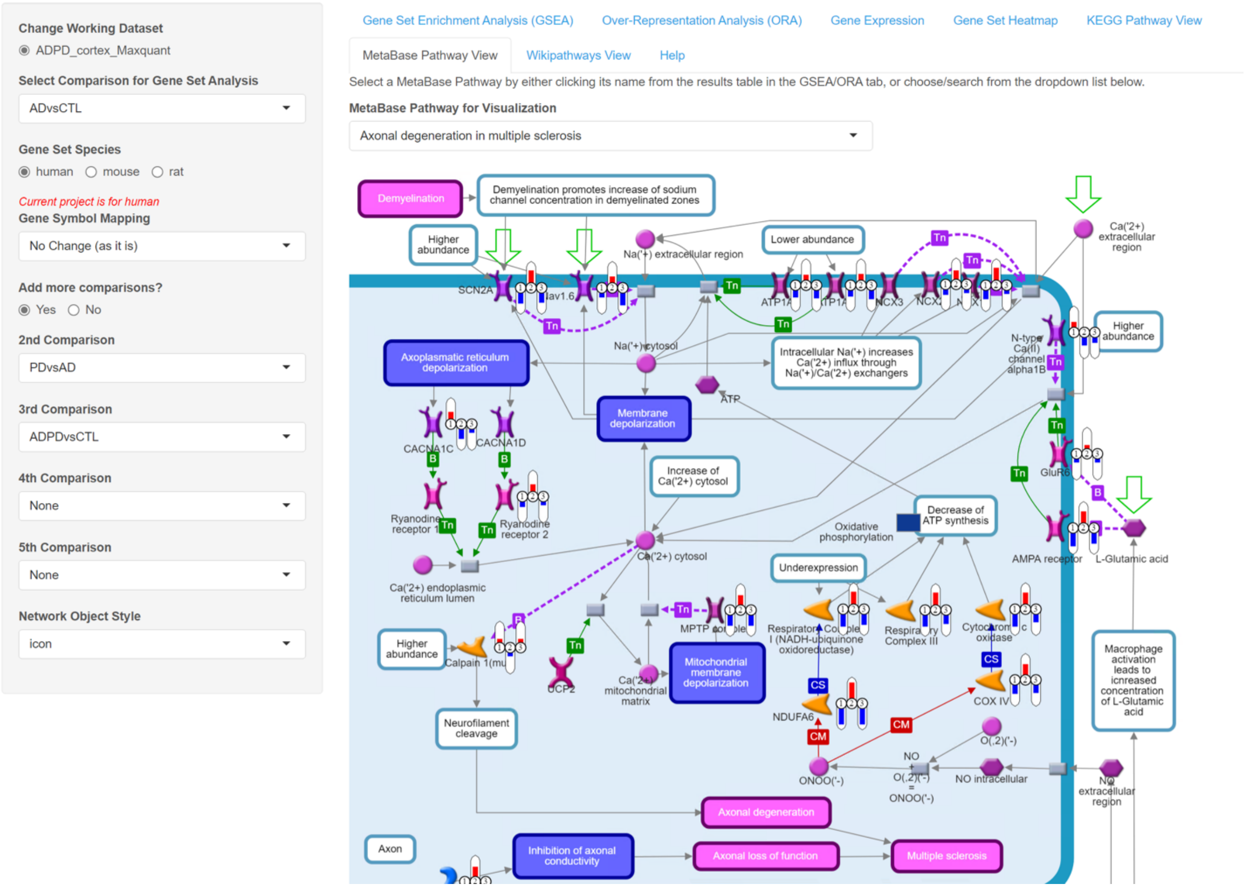

Figure S11: Overlay of data on Metabase pathway diagrams. This figure illustrates that the Metabase pathway maps are fully compatible with xOmicsShiny. Users may need to request a license to access the Metabase pathway database.

Dataset	Samples	Comparisons	Measurements	Loading (s)	GSEA calculation (s)				ORA calculation (s)			
					KEGG	GO, BP	GO, CC	GO, MF	KEGG	GO, BP	GO, CC	GO, MF
Mouse Microglia RNA	93	9	15,402	9.2	3.8	17.1	8.1	8.1	1.3	15.7	2.7	3.7
LRRK2 Neuron RNA	54	2	15,168	7.1	7.8	24.3	6.4	10.2	1.7	19.1	3.3	4.7
LRRK2 Neuron Proteome	76	2	9,807	7.2	4.5	22.8	16.2	9.0	1.5	15.7	3.1	3.9
ADPD cortex Searched by Maxquant	49	6	7,163	5.3	2.9	7.8	4.4	4.2	1.0	12.1	2.4	3.1
ADPD ACG Searched by Maxquant	50	6	8,145	5.4	5.9	8.5	4.6	4.5	1.1	13.1	2.3	3.3
ADPD cortex Searched by PD	49	6	8,746	5.5	3.6	11.1	6.1	4.9	1.2	12.6	2.1	3.2
ADPD ACG Searched by PD	50	6	9,605	5.5	3.6	10.3	5.9	5.1	1.1	12.2	2.1	3.4
Aging HFCD Metabolomics	590	4	393	4.6	1.4	NA	NA	NA	1.1	NA	NA	NA
Aging HFCD Proteomics	315	4	3,941	6.5	3.3	7.2	4.1	3.9	1.1	12.1	2.1	3.1
Aging HFCD RNAseq	291	4	25,393	21.3	5.4	37.5	9.4	9.4	1.6	19.8	2.9	4.6
Stroke Brain Time Course	70	NA	7,565	7.1	NA	NA	NA	NA	NA	NA	NA	NA
LCM 96Grid Simulated	96	1	3,881	5.4	2.7	7.3	4.3	3.8	1.1	11.3	1.9	3.1

Table S1: Running speed test of example datasets. This table shows the number of samples, comparisons, and measurements (genes/proteins/metabolites) in each dataset. The additional columns record the loading speed (in seconds) of the data, as well as the time to calculate GSEA (Gene set enrichment analysis) and ORA (Over-representation analysis) results using the KEGG or gene ontology (GO) databases (BP: biological process, CC: cellular component, and MF: molecular function).

Main features		xOmicsShiny	Quickomics	Holomics	GraphBio	MiBiOmics	EasyPubPlot
Single-omics	General analysis	✓	✓	✓	✓	✓	✓
	PCA	✓	✓	✓	✓	✓	✓
	Volcano plot	✓	✓		✓		✓
	Heatmap	✓	✓	✓	✓		✓
	Expression boxplot	✓	✓				✓
	Venn Diagram	✓	✓				
	Correlation analysis	✓	✓	✓		✓	
	Network analysis (WGCNA, etc.)	✓			✓	✓	
	GSEA/GO analysis	✓	✓		✓		✓
	Pattern and trend analysis	✓					
Multi-omics	Multi-omics integration	✓		✓		✓	
	Multi-omics data exploration	✓		✓		✓	
	Multi-omics volcano plot	✓					
	Multi-omics pathway mapping	✓					
Citation		Current work	Quickomics: exploring omics data in an intuitive, interactive and informative manner, Bioinformatics	Holomics-a user-friendly R shiny application for multi-omics data integration and analysis, BMC Bioinformatics	GraphBio: A shiny web app to easily perform popular visualization analysis for omics data, Frontiers in Genetics	MiBiOmics: an interactive web application for multi-omics data exploration and integration, BMC Bioinformatics	EasyPubPlot: a shiny web application for rapid omics data exploration and visualization, bioRxiv

Table S2: Feature comparison with existing R Shiny applications.
